# Editorial: recent discoveries in evolutionary and genomic microbiology

**DOI:** 10.3389/fmicb.2015.00323

**Published:** 2015-04-16

**Authors:** Anton G. Kutikhin, Arseniy E. Yuzhalin

**Affiliations:** ^1^Laboratory for Genomic Medicine, Division of Experimental and Clinical Cardiology, Research Institute for Complex Issues of Cardiovascular DiseasesKemerovo, Russia; ^2^Cancer Research UK/MRC Oxford Institute for Radiation Oncology, Department of Oncology, University of OxfordOxford, UK

**Keywords:** human microbiome, gut microbiome, oral microbiome, human ecology, evolution, domains of life, human mycobiome, brain-gut-microbe axis

According to the current knowledge, the human body can be viewed as a superorganism comprised of human cells and resident microbial communities called microbiota (Ley et al., 2008). Human microbiota has a wide range of physiological functions, playing an important role in digestion, immunity, and production of certain vitamins; however, most of these microorganisms cannot be cultured in laboratory conditions, and until recently it has been the main challenge for clear determination of the composition of human microbiome (Tlaskalová-Hogenová et al., 2011). New discoveries in methods of molecular biology, including the emergence of omics technologies, have provided a possibility for deciphering the qualitative and quantitative composition of the microbiome (Tlaskalová-Hogenová et al., 2011). This breakthrough has also stimulated in-depth investigation of host-microbiota interactions (Tlaskalová-Hogenová et al., 2011). Basic comparative research revealed that each individual has a unique microbiota, which composition is largely determined during the first years of life but can be altered, reversibly or irreversibly, by a number of factors, such as age, environment, diet, drugs, diseases, and others (Zoetendal et al., 2008).

Therefore, the question of human microbiome biology is extremely intriguing, and large amount of research has been published during the recent years. The investigation of gut, oral, respiratory, skin, vaginal, urinary microbiomes is gaining increasing interest and more attention with time (Sommer and Bäckhed, 2013; Belkaid and Segre, 2014; Xu and Gunsolley, 2014; van de Wijgert et al., 2014; Rogers et al., 2015; Shreiner et al., 2015; Whiteside et al., 2015). We have opened this Research Topic with the idea to provide an outlook for discoveries that have become milestones in the field.

We sincerely thank all researchers who have agreed to contribute to our Research Topic. This collection is divided into three sections. The first one includes three articles in which Eric Bapteste, Christopher House, Matteo Pellegrini, and Sorel Fitz-Gibbon discuss the general issues of microbial evolution whilst Arshan Nasir, Patrick Forterre, Kyung Mo Kim, and Gustavo Caetano-Anolles analyze the distribution of viruses and their impact on evolution of organisms.

The second section is devoted to the biology of human microbiome. We highly recommend an excellent article of Sara Quercia and colleagues who describe the timescales of human gut microbiota adaptation and talk over gut microbiota plasticity that ≪was strategic to face changes in lifestyle and dietary habits along the course of the recent evolutionary history, that has driven the passage from Paleolithic hunter-gathering societies to Neolithic agricultural farmers to modern Westernized societies≫. The article by Noah Voreades, Anne Kozil, and Tiffany Weir extends this topic, focusing primarily on diet as ≪one of the most pivotal factors in the development of the human gut microbiome from infancy to the elderly ≫. Readers interested in biology of aging will appreciate the paper by Sitaraman Saraswati and Ramakrishnan Sitaraman on the causative role of gut microbiota in this process. As immune and neuroendocrine systems maturate together with gut microbes, we are glad to present a review by Sahar El Aidy, Timothy Dinan, and John Cryan who comprehensively analyze this topic. Gut microbiota also plays a major role in non-communicable diseases, for example, obesity and type 2 diabetes mellitus, and Isabel Moreno-Indias, Fernando Cardona, Francisco Tinahones along with Maria Isabel Queipo-Ortuno present a good story on it. Regarding original research and methods, Eamonn Culligan, Julian Marchesi, Colin Hill, and Roy Sleator demonstrate how the combination of metagenomic and phenomic approaches helps us to identify novel genes within the gut microbiome. Further, Hui Chen and Wen Jiang discuss the role of the oral microbiome in health and diseases. At last, there are several papers on the human mycobiome, and Ablishek Saxena along with Ramakrishnan Sitaraman shed light on the aspects of its osmoregulation.

The last piece of the collection is composed by articles on other topics. Milton Saier and Zhongge Zhang discuss an intriguing principle of directed mutation, Ramakrishnan Sitaraman talks about *Helicobacter pylori*, DNA methyltransferases and the epigenetic field effect in cancerization, and Anton Kutikhin with colleagues summarize recent discoveries on calcifying nanoparticles sometimes referred to as nanobacteria.

We created this Research Topic with the hope that it will be useful for a wide audience, particularly immunologists, microbiologists, graduate, and undergraduate students of biomedical faculties as well as their lecturers.

## Conflict of interest statement

The authors declare that the research was conducted in the absence of any commercial or financial relationships that could be construed as a potential conflict of interest.
